# Demonstrative Midwifery

**Published:** 1851-03

**Authors:** 


// THE WESTERN JOURNAL.

THIRD SERIES.—VOL. VII.—NO. III.

LOUISVILLE, MARCH 1, 1851.

DEMONSTRATIVE MIDWIFERY.

The readers of Medical Journals are familiar with the clamor raised about twelve months since, by a band of medical men, in Buffalo, New York, on the subject of Demonstrative Midwifery. After the trial of Dr. Loomis, for libel, a report of the trial was published and distributed among the medical profession, and a copy of the pamphlet fell into the hands of a captious correspondent of the American Journal of the Medical Sciences. This snarling critic holds that he has reached the ultimathule of obstetrical science, and that he is, not only consequentially, but consequently infallible. He gravely assumes that those who differ from him are necessarily in the wrong, and he boldly denounces Professor White for managing an obstetrical case in accordance with his own good judgment, and with many eminent authorities. We cannot understand how the captious gentleman to whom we refer, can reconcile his treatment of Professor White with the principles of any sound code of medical ethics known to medical men.

The able and worthy editor of the American Journal of the Medical Sciences has very properly repudiated the judgment of his correspondent, and acknowledges that Dr. White's treatment of his case was not open to the cavils of the critic. And the judgment of nine-tenths of the American medical profession, who have read the paltry diatribe of the Philadelphia critic, will find no difficulty in coming to the conclusion, that the critic in attempting to write down Professor White, succeeded admirably in writing himself down. While we look with regret upon any attempt on the part of medical men to put down a member of the profession for an honest discharge of his duties, we rejoice that Dr. White has been amply vindicated, and triumphantly sustained.

In a recent number of the Buffalo Medical Journal, Professor Flint has, in his usually efficient and masterly manner, vindicated his absent colleague from the false and improper aspersions that were thrown upon him by one who claims to belong to a noble, liberal and highminded profession. Professor Flint has done ample justice to the subject, and we thank him for the stand he has taken in favor of improvements in teaching medicine. We look upon every real advancement of the means of medical education, every real increase of the resources of medical instruction, as increased means of benefit to mankind, and among these increased resources we regard Demonstrative Midwifery worthy of a high place. We have seen and heard enough to know that demonstrations in that department of medical education are absolutely essential, and the only regret we feel in connection with the subject, is that the matter has too many inherent difficulties, without the aid of medical cavillers.

The editor of the American Journal of the Medical Sciences states that the judgment of the medical profession in Philadelphia is adverse to demonstrative midwifery. In common with Professor Flint, we were surprised at that statement, for we happen to know that some gentlemen who are regarded as quite eminent among the medical men of Philadelphia have expressed quite a different view from the one assumed by the editor of the Philadelphia Medical Journal. But, taking the statement of the editor for granted, we think, even if the medical men of Philadelphia were as unanimous as they are represented to be on this subject, that it would not be the first time in their history that they were in the wrong, They were nearly, if not quite unanimous in opposition to Dr. Shippen's effort to introduce Obstetrics into the hands of medical men. That was once denounced quite as bitterly in Philadelphia as Dr. White's critic denounces him, and we entertain no doubt that the wiseacres who opposed Dr. Shippen felt well assured that they were the light of the earth. The men who should now undertake to advocate the views of Dr. Shippen's opponents would run the risk of being looked upon as arrant quacks.

We feel that we owe these remarks to the character of a medical gentleman who has honestly endeavored to benefit his profession, and we are sure that he will be sustained by all who have the true interests of medical science at heart. B.

---

☞ Several editorial and other articles, prepared for this number of the Journal, are unavoidably deferred.

